# Colonic stem cells from normal tissues adjacent to tumor drive inflammation and fibrosis in colorectal cancer

**DOI:** 10.1186/s12964-023-01140-1

**Published:** 2023-08-01

**Authors:** Yuanyuan Zhao, Mengmeng Guo, Fuqiang Zhao, Qian Liu, Xia Wang

**Affiliations:** 1 School of Pharmaceutical Sciences, Tsinghua University, Beijing, 100084 China; 2 Institute of Biomedical Engineering, Chinese Academy of Medical Sciences and Peking Union Medical College, Tianjin, 300192 China; 3 Department of Colorectal Surgery, National Cancer Center, National Clinical Research Center for Cancer, Cancer Hospital, Chinese Academy of Medical Sciences and Peking Union Medical College, Beijing, 100021 China

**Keywords:** Stem cells, Colorectal cancer, Microenvironment, Fibrosis, Inflammation

## Abstract

**Background:**

In colorectal cancer (CRC), the normal tissue adjacent to tumor (NAT) communicates actively with the tumor. Adult stem cells from the colon play a crucial role in the development of the colonic epithelium. In the tumor microenvironment, however, it is unclear what changes have occurred in colonic stem cells derived from NAT.

**Methods:**

Using an intestinal stem cell culture system, we cultured colonic cells from NAT and paired CRC tissue, as well as cells from healthy tissue (HLT). Clonogenicity and differentiation ability were used to compare the function of clones from NAT, HLT and CRC tissues. RNA high-throughput sequencing of these clones was used to identify the molecular characteristics of NAT-derived clones. Coculture of clones from HLT and CRC was used to assess molecular changes.

**Results:**

We found that the morphological characteristics, clonogenic ability, and differentiation ability of NAT-derived clones were consistent with those of HLT-derived clones. However, NAT-derived clones changed at the molecular level. A number of genes were specifically activated in NAT. NAT-derived clones enriched pathways related to inflammation and fibrosis, including epithelial mesenchymal transition (EMT) pathway and TGF-beta signaling pathway. Our results also confirmed that NAT-derived clones could recruit fibroblasts in mice. In addition, HLT-derived clones showed high expression of FOSB when cocultured with tumor cells.

**Conclusions:**

Our results demonstrate that colonic stem cells from NAT in the tumor microenvironment undergo changes at the molecular level, and these molecular characteristics can be maintained in vitro, which can induce fibrosis and an inflammatory response.

Video Abstract

**Supplementary Information:**

The online version contains supplementary material available at 10.1186/s12964-023-01140-1.

## Introduction

CRC is currently one of the most prevalent malignant tumors worldwide and the second-leading cause of cancer-related death [1]. Recent developments in tumor biology have shown that understanding the various underlying mechanisms of tumor progression requires a comprehensive review of the complex exchanges between tumor cells and their adjacent microenvironment [2, 3]. Tumors and NAT can interact effectively with one another and with other cell types. When comparing tumor tissue to normal tissue, we frequently classify tissue with normal morphology as normal tissue. However, histologically normal tissue is not truly "normal” and can be morphologically normal but molecularly altered to a preneoplastic state [4]. In transcriptome investigations of breast cancer [5], prostate cancer [6], colon cancer [7], and gastric cancer [8], a distinct gene expression profile of NAT has been discovered. Despite the fact that NAT includes a variety of cell types, to our knowledge, no research has been done on a particular cell type in NAT. Additionally, colonic stem cells are necessary for the development of the epithelial structure in the colon, and are usually considered as the original cells of CRC [9–11]. It is unclear what changes have taken place in stem cells in the tumor microenvironment.

We previously established an effective and long-lasting culture of 2D human colonic stem cells by optimizing a 3T3 co-culture system [12–14]. We cultured cancer cells from CRC tissue, as well as colonic stem cells from NAT and HLT, using this technique. The overall RNA expression profile of clones derived from these various tissues was evaluated through deep sequencing. Our results demonstrated that colonic stem cells in tumor microenvironment could be stimulated by adjacent tumor tissue to undergo molecular changes and induce fibrosis and inflammatory responses. Additionally, we discovered proteins involved in cancerization [15, 16]. These dysregulated proteins provide a novel molecular mechanism for the biology of field cancerization and can be used as biomarkers for tumor diagnosis. Despite having comparable histological appearances, molecular characteristics could distinguish that set apart NAT-derived clones from HLT-derived clones. Some of the molecular characteristics of NAT are oncogenic, which may explain why CRC survivors are more likely to develop metachronous malignancies and suggests possible targets for posttreatment surveillance.

## Materials and methods

### Human Samples

This study was approved by the Ethics Committee of the Institution Review Board of Tsinghua University (#20,170,019 and #20,190,303), National Cancer Center/Cancer Hospital, Chinese Academy of Medical Sciences, and Peking Union Medical College (#19/172–1956). NAT and CRC tissue samples were obtained from patients who were diagnosed with CRC and underwent surgical resection at the hospital. HLT samples were collected from normal biopsy samples of a patient with benign colon polyps and another with Tics and Tourette syndrome (Tic syndrome). HLT samples are collected from three different parts of each patient. All patients gave written informed consent. Information on cancer and noncancer tissue specimens is shown in the Supplementary Table S1.

### In vitro culture of primary cells from NAT, HLT and tumor tissues

All tissues were excised after surgery, stored and transported in wash buffer: F12 (Gibco), 5% FBS (Hyclone), 1% penicillin/streptomycin (Gibco), 0.1% Amphotericin B (Gibco), 0.25% Gentamicin (Gibco), 1% HEPES (Gibco), and 5 μM Rock inhibitor (Calbiochem) at 4 °C. All tissues were cut into small pieces and incubated in 1 mg/ml collagenase type XI buffer (Gibco) at 37 °C, but incubation time was different. 15 min for tumor tissue, 60 min for NAT and HLT. The digested cell solution was filtered through a 70 μm cell strainer (Falcon), and washed four times with a wash buffer. Isolated cells were resuspended in stem cell medium (SCM): Advanced DMEM/F12 (Hyclone) supplemented with 10% FBS, 1% penicillin/streptomycin, 1% L-Glutamine (Hyclone), 0.1% Amphotericin B, 0.5% Gentamicin, 0.18 mM Adenine (Sigma), 5 μg/mL Insulin (Sigma), 2 nM T3 (Sigma), 200 ng/mL Hydrocortisone (Sigma), 125 ng/mL R-Spondin 1(R&D), 1 μM Jagged-1(AnaSpec Inc), 100 ng/mL Noggin (Peprotech), 2.5 μM Rock inhibitor (Calbiochem), 2 μM SB431542 (Cayman chemical), 10 mM Nicotinamide (Sigma), and 10 ng/mL EGF (Upstate Biotechnology). After resuspension, the cells were seeded into irradiated 3T3-J2 feeder cells that were paved one day in advance, and cultured at 37 °C in 7.5% CO_2_. The culture medium was replaced every two days.

### In vitro differentiation and feeder removing

The air–liquid interface (ALI) culture of stem cell clones was performed as described [12]. The colonies were digested in a 0.25% trypsin–EDTA solution at 37 °C for 8 min and passed through 30 µm filters (Miltenyi Biotec) to obtain single cells. The pellets were resuspended in 80 μL F12 and 20 μL mouse feeder removing beads (Miltenyi Biotec) and incubated at 4 °C for 15 min to avoid light. Each Transwell insert (Corning) was coated with 20% Matrigel (growth factor reduced, BD Biosciences) and incubated at 37 °C for 30 min to polymerize. 200,000 feeder cells were seeded into each transwell insert and incubated overnight at 37 °C in 7.5% CO_2_. Placed columns in the magnetic MACS Separator and rinsed with 3 mL F12 buffer. Then 400 μL F12 to the cells were added before passing through the columns. Finally, colonies were eluted with another 2 mL F12. 200,000—300,000 cells were seeded into each transwell insert and cultured with SCM medium. After reaching confluency (3—7 days), the top medium was removed by carefully pipetting, and the cells were further incubated for 6—12 days in SCM minus nicotinamide medium (SCM-6) before analysis. The SCM-6 medium was changed every day.

### Immunohistochemistry and Immunofluorescence

Sections of formalin-fixed, paraffin-embedded tissues, xenografts and ALI tissues were stained by standard HE staining. For immunohistochemistry and immunofluorescence staining, slides were subjected to antigen retrieval in citrate buffer (pH 6.0, Sigma-Aldrich) at 95 °C for 20 min, and a blocking procedure was performed overnight with 5% bovine serum albumin (BSA, Sigma-Aldrich,) and 0.05% Triton X-100 (Sigma-Aldrich) in DPBS(-) (Gibco) at 4 °C. Primary antibodies used in this study included antibodies against MUC2 (Santa cruz biotechnology, 1:200) and E-Cadherin (R&D Systems, 1:200), Vimentin (Cell Signaling Technology, 1:200), CD45(Invitrogen, 1:500), Chromogranin A (Abcam, 1:200), Villin (Cell Signaling Technology, 1:200). Secondary antibodies were either Alexa Fluor-488 or Alexa Fluor-594 Donkey anti-goat/mouse/rabbit IgG (Thermo Fisher Scientific). Images were acquired by Olympus IX73 microscopy.

### Western blot

The colonies were collected in RIPA buffer (Thermo Fisher Scientific) containing protease inhibitor cocktail (Bimake) and phosphatase inhibitor cocktail (1:100, Bimake). Protein was quantified with BCA reagent (Thermo Fisher Scientific). The extracts were resolved by SDS-PAGE on a 10% gradient gel, transferred onto PVDF membranes (GE healthcare life sciences), and incubated with antibodies against FOSB (Cell Signaling Technology, 1:1000), GAPDH (Cell Signaling Technology, 1:1000), EDIL3(Santa cruz biotechnology,1:1000) overnight at 4 °C. Incubation with secondary antibody (Anti-mouse IgG, HRP-linked Antibody; Cell Signaling Technology) was performed for 2 h at RT. After detection using an ECL western blot substrate (Thermo Fisher Scientific), images were acquired using an ImageQuant LAS 4000.

### Subcutaneous injection

Mice aged 6 to 8 weeks were maintained under pathogen-free conditions, in accordance with Tsinghua University Animal Ethics Committee guidelines. After removing feeder cells, colonies were harvested and subcutaneously injected into NSG (NOD.Cg-Prkdc^scid^ Il2rg^tm1^) mice at 10^6^ cells in 100 μL 50% Matrigel. Each clone from one patient sample was injected into five mice. Animals were sacrificed with CO_2_ after 4 weeks.

### RNA-seq analysis

After removing feeder cells, RNA was isolated from colonies with Trizol Reagent (Invitrogen) and with RNeasy mini kit (QIAGEN). 2 µg of prepared input RNA was sequenced on the Illumina platform generating 150 bp paired-end reads. Gene abundance was quantified using HISAT2 [17] (version 2.0.5) and StringTie [18] (version 1.3.4) pipeline [19]. Alignment results were then input to DEseq2 (version 1.24.0) [20] for differential expression analysis at a strict threshold of adjusted *p* < 0.01 and |log2(fold-change)|> 1. The heatmaps with hierarchical clustering analysis of the global gene expression pattern in different samples were performed using pheatmap package [21] in R.

Gene set enrichment analysis (GSEA) and Gene Ontology term enrichment analysis (GOEA) were performed with R package clusterProfiler (version 3.12.0) [22]. Principal Component Analysis (PCA) was performed based on top 10,000 most variable genes by prcomp function in R and was visualized with ggbioplot (version 0.55) (https://github.com/vqv/ggbiplot).

### Whole-exome sequencing (WES) analysis

After removing feeder cells, genomic DNA was extracted with DNeasy Blood & Tissue kit (Qiagen). 1–3 µg DNA was used for WES. Single base mutations, insertions, and deletions were identified by first aligning the Illumina pair-end reads to the mouse reference genome (GRCm38), and using Bowtie2 (version 2.3.2) to remove the contamination DNA of mouse cells from feeder layer. Then, filtered reads were quality-filtered by trim galore, aligned to human reference genome (hg19) by BWA (version 0.7.15) [Aligning sequence reads, clone sequences and assembly contigs with BWA-MEM] and transfer to BAM files by Samtools (version 1.3.1) respectively [23]. Next, Picard (version 2.18.27) was used to mark duplicate reads from BAM files, and Genome Analysis Toolkits (version 4.1.0.0) was used to recalibrate with default parameters. Mutations in NAT samples and CRC samples were called by comparison to reference genome using Mutect2. Variants were annotated to the functional consequence using ANNOVAR (version Apr 2018) based on human genome build hg19 [24].

### Statistical analysis

Analysis procedures of transcriptome data were provided in the relevant sections of Method Details. Statistical analysis was performed with GraphPad Prism and presented as mean values ± SD. Unpaired two-tailed Student’s t test was used to calculate the p values between the two groups. Corresponding statistical significance was denoted with (∗ *p* < 0.05; ∗∗ *p* < 0.01; ∗∗∗ *p* < 0.001 and ∗∗∗∗ *p* < 0.0001) in the figures and figures legends.

## Results

### NAT-derived clones exhibit normal morphological traits

**Fig. 1:**
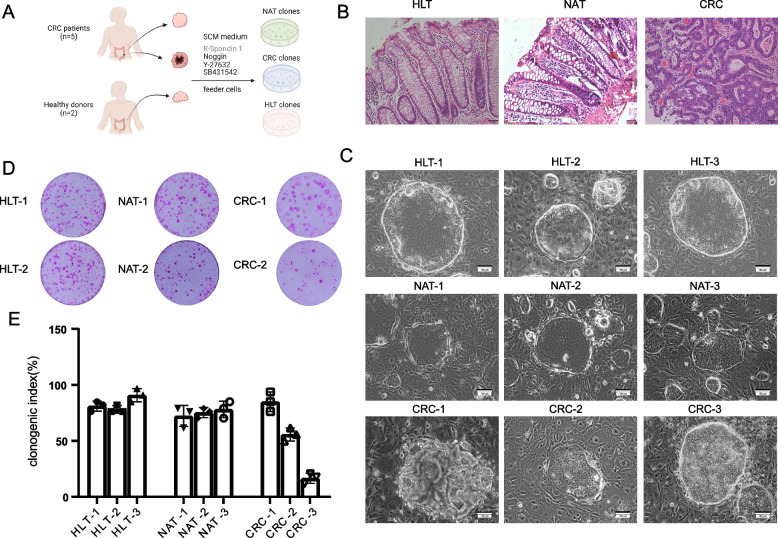
Establishment of clones derived from NAT, HLT and tumor tissues. **A** Overview of experimental design. The clones were derived from NAT and paired CRC tissues, as well as clones from HLT. **B** Representative H&E staining images of the original tissues. Scale bar, 50 µm. **C** Representative bright field microscopy images of clones from NAT, HLT and CRC tissues on lawns of irradiated 3T3-J2 feeder cells. Scale bar, 100 µm. **D** Rhodamine staining showing the clonogenicity assay of colonies grown on feeder cells following seeding 1,000 single cells per well from each clone derived from NAT, HLT and CRC tissues. **E** Histogram of clonogenicity index based on percentage of plated cells that form colonies. Data represented as mean ± SD; *n* = 3 technical replicates

Using irradiated 3T3-J2 murine embryonic fibroblasts as the feeder cell layer, we cultured the colonic stem cell clones from NAT and corresponding tumor samples from five patients with colorectal cancer, as well as HLT from two nontumor bearing patients (Fig. 1A, B). All clones were grown on irradiated 3T3-J2 feeder cells.

Although NAT and HLT could produce 2000 to 3000 independent clones, the tumor tissues differed significantly. Typically, 100 ~ 10,000 distinct clones could be produced. The morphology of NAT-derived clones was identical to that of healthy tissue clones. However, there were significant morphological differences between tumor tissue-derived clones, such as large vacuoles and solid spheres (Fig. 1C). Additionally, we tested the clonogenic ability of NAT-derived and HLT-derived clones and found no difference. The clonogenicity of clones from NAT and HLT were comparable, at approximately 76.1% ± 2.52% and 82 ± 2.67%, respectively (Fig. 1D, E). The clonogenic ability of clones from CRC tissues varied from person to person. As showed in our previous study, we would like to emphasize that the clonogenic ability of clones derived from CRC tissues can vary depending on the gene mutation background of primary tumor cells and the dependence on various growth factors [14].

### NAT-derived clones can normally differentiate

**Fig. 2:**
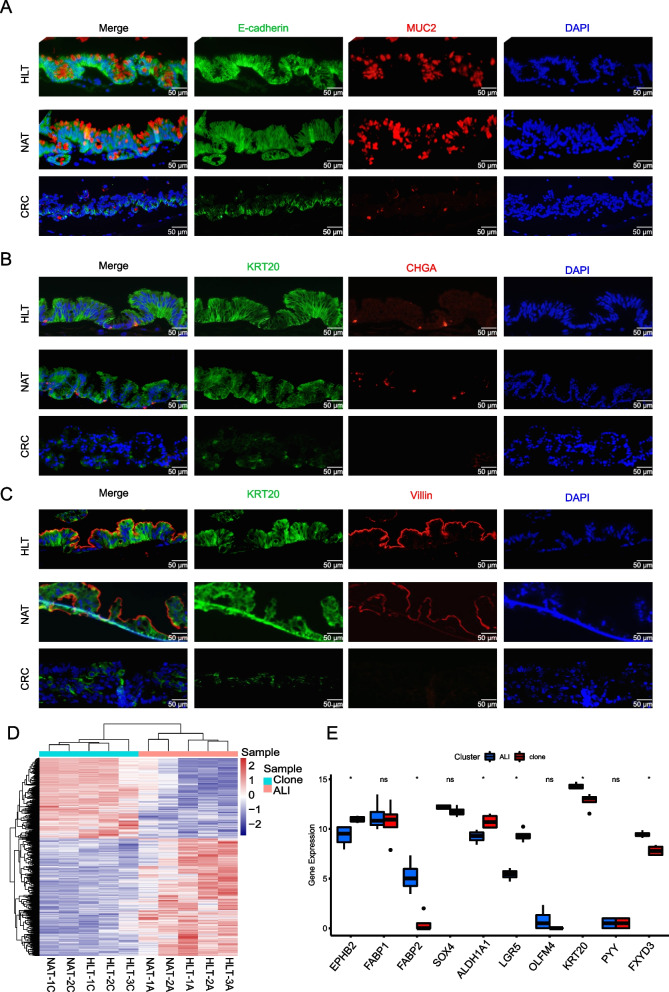
Differentiation capacity of clones derived from NAT, HLT and tumor tissues. **A** Immunofluorescence images of ALI mini-guts stained with E-cadherin (green) and Muc2 (red). Nuclei (blue) were stained with DAPI. Scale bar, 50 μm. **B** Immunofluorescence images of ALI mini-guts stained with KRT20 (green) and CHGA (red). Nuclei (blue) were stained with DAPI. Scale bar, 50 μm. **C** Immunofluorescence images of ALI mini-guts stained with KRT20 (green) and Villin (red). Nuclei (blue) were stained with DAPI. Scale bar, 50 μm. **D** Heatmap shows normalized gene expression of stem cell clones (Clone) on feeder cells and corresponding ALI mini-guts (ALI). **E** Gene expression of stemness markers in stem cells clones and corresponding ALI mini-guts based on RNA-seq data

We demonstrated that intestinal stem cells could differentiate into normal epithelia using the ALI system in vitro [12]. Under ALI conditions, intestinal stem cells form a 3D structure called ALI mini-guts in two to three weeks, and the ALI mini-guts could be maintained for 17–30 days. To assess whether NAT-derived clones can normally differentiate similar to healthy tissues, we planted clones on the ALI to form ALI mini-guts and performed immunofluorescence staining. The results revealed that the ALI mini-gut generated from both NAT and HLT maintained certain polarity characteristics, and differentiated cells expressed mucin 2 (MUC2, maker of goblet cells), chromogranin A (CHGA, maker of endocrine cells) and polarized villin expression (Fig. 2A, B, C). The 3D structure generated from tumor cells was different since their polarity characteristics were not obvious, and the markers of mature cells were hardly expressed.

The heatmap showed that clones on feeder cells and corresponding ALI mini-guts from NAT and HLT differed dramatically in gene expression (Fig. 2D). The clones showed high expression of intestinal stem cell markers such as *EPHB2*, *ALDH1A1*, *LGR5* [25, 26], while the differentiation-related maker *KRT20, FABP2, FXYD3* were highly expressed in ALI mini-guts (Fig. 2E).

### NAT clones are distinct from clones derived from both healthy and tumor tissues

**Fig. 3:**
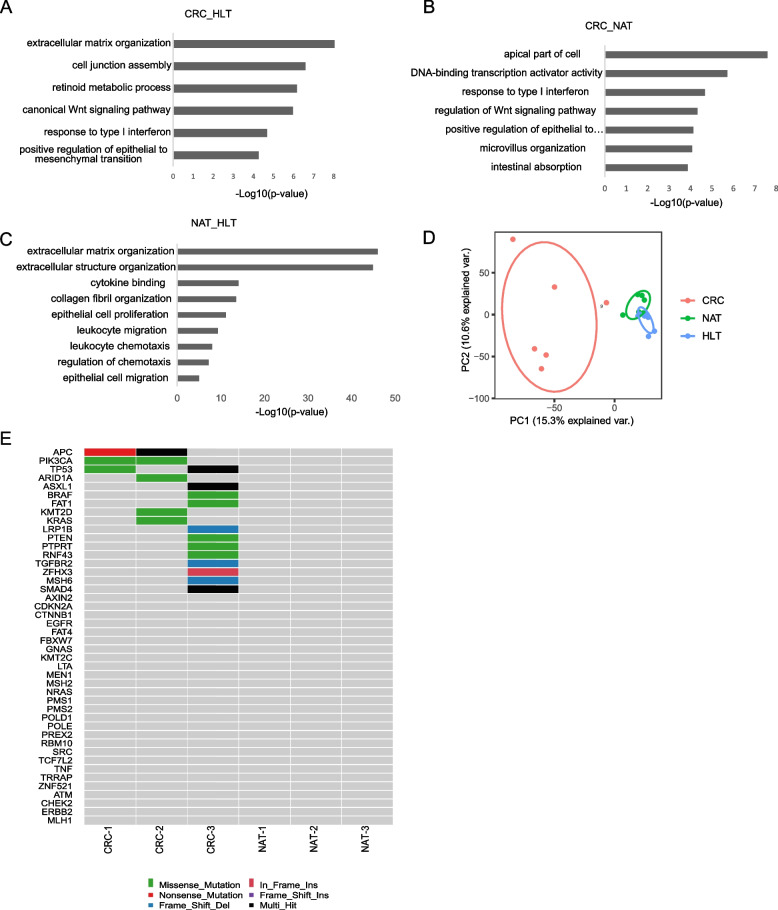
Gene expression differences between clones derived from NAT, HLT and tumor tissues. **A** Bar plots of the enriched GO terms of upregulated genes in CRC tumor tissues compared with HLT. The terms were selected from the top 20 biological process terms, *p* < 0.001. **B** Bar plots of the enriched GO terms of upregulated genes in CRC tumor tissues compared with NAT. The terms were selected from the top 20 biological process terms, *p* < 0.001. **C** Bar plots of the enriched GO terms of upregulated genes in NAT compared with HLT. The terms were selected from the top 20 biological process terms. **D** The principal components analysis (PCA) representing the dispersion of the samples based on their gene expression levels. CRC clones (red), NAT clones (green) and HLT clones (blue). **E** Somatic mutations found in CRC related genes are present in CRC clones but not in NAT clones. Mutation type is indicated in the legend

To characterize the molecular characteristics of NAT, we performed differentially expressed genes (DEGs) analysis of NAT-derived clones, corresponding tumor cells, and HLT-derived clones. By comparing tumor cells with HLT clones, we identified 1399 upregulated and 805 downregulated DEGs. To better understand the transcriptome differences between these groups, we performed the GO term analysis based on differentially expressed genes and selected the most significant signatures (*p* < 0.001), such as extracellular matrix organization (*p* = 9.11E^−09^) and canonical Wnt signaling pathway (*p* = 5.91E^−07^), and cell junction assembly (*p* = 2.51E^−07^; Fig. 3A). By comparing NAT clones with corresponding tumor cells, we identified 810 upregulated and 766 downregulated DEGs. GO enrichment was used to identify 178 categories (*p* < 0.05), such as the apical part of the cell (*p* = 2.55E^−8^), type I interferon signaling pathway (*p* = 3.18E^−5^), and the regulation of epithelial to mesenchymal transition (EMT, *p* = 7.45E^−5^; Fig. 3B). We also assessed DEGs between NAT clones and HLT clones, and found 1096 DEGs (723 upregulated and 373 downregulated; Supplemental Table S2). Remarkably, among the interesting GO categories, extracellular matrix organization (*p* = 8.58E^−47^) and the BMP signaling pathway (*p* = 4.38E^−5^) appeared to be exclusive to the comparison group between NAT and HLT, while cell chemotaxis (*p* = 1.99E^−11^) and the regulation of EMT pathway (*p* = 1.72E^−08^) emerged from both comparisons (Fig. 3C).

Through principal component analysis, we discovered that the three tissue derived clones were distinct from one another, and the NAT clones were between healthy clones and tumor cells (Fig. 3D). NAT was in an intermediate molecular state between healthy and malignant tissue. Consistent with the histologic appearance, NAT samples had a closer relationship to the HLT samples than to the tumor samples.

In addition, to rule out the possibility that the clones derived from NAT have become cancerous, whole exome sequencing was performed on three NAT derived clones and three CRC tissues-derived clones. The sequencing results revealed that the NAT derived clones did not have the most common mutations during colorectal cancer processes (Fig. 3E).

### NAT-derived clones highly express inflammatory related genes

**Fig. 4:**
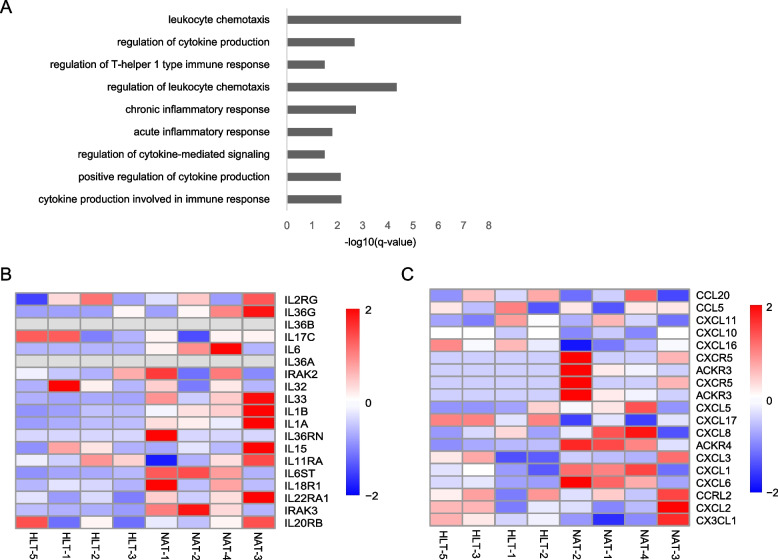
Inflammatory gene expression by NAT derived clones. **A** Bar plots of the enriched GO terms of inflammation related genes in clones derived from NAT compared with HLT. **B** Differential expression heatmaps of interleukin genes among RNA-seq DEGs (FDR < 0.05) of clones derived from NAT and HLT. **C** Differential expression heatmaps of chemokine genes among RNA-seq DEGs (FDR < 0.05) of clones derived from NAT and HLT

Using pathway enrichment analysis to generate gene expression profiles of the clones from NAT and healthy tissues, we found that NAT clones were enriched in multiple inflammatory pathways, including interferon-gamma production, leukocyte chemotaxis, positive regulation of cytokine production, regulation of T-helper 1 type immune response, and chronic inflammatory response (Fig. 4A). A more detailed expression heatmap revealed that NAT-derived clones exhibited constitutive expression of a broad array of chemokines and interleukins genes compared to HLT-derived clones. In addition, NAT-derived clones expressed a broad array of interleukin genes, including *IL33, IL1B, IL1A, IL6ST, IL18R1, IL22RA1* and *IRAK3* (Fig. 4B). Among these chemokines were key determinants of neutrophil responses, including *CXCL1, CXCL6*, and *CXCL8* (Fig. 4C). Multiple cellular mediators are known to interact with innate and adaptive immune responses. It is noteworthy that the inflammatory gene expressed in the clones isolated from NATs will continue to be activated for several months even in a sterile in vitro environment.

To assess the pathogenic potential of NAT clones, we subcutaneously injected NAT clones from three CRC patients and three control patients into highly immunodeficient NSG (NOD^scid^IL2ra^null^) mice [27]. One million cells of a designated clone were mixed with 50% Matrigel and subcutaneously injected, and the resulting xenograft nodule was examined four weeks later. All clones were assembled into a polarized epithelium in vivo. Unlike the grafts formed by HLT clones, NAT clones have leukocyte infiltration as evidenced by anti-murine CD45 staining, similar to CRC tissues derived clones (Fig. S1). These findings suggest that NAT clones express highly inflammatory related genes compared to HLT clones. Further investigation, using humanized immune system mouse models, would demonstrate whether these NAT-derived clones can recruit immune cells."

### Xenografts of NAT-derived clones drive fibrosis

**Fig. 5:**
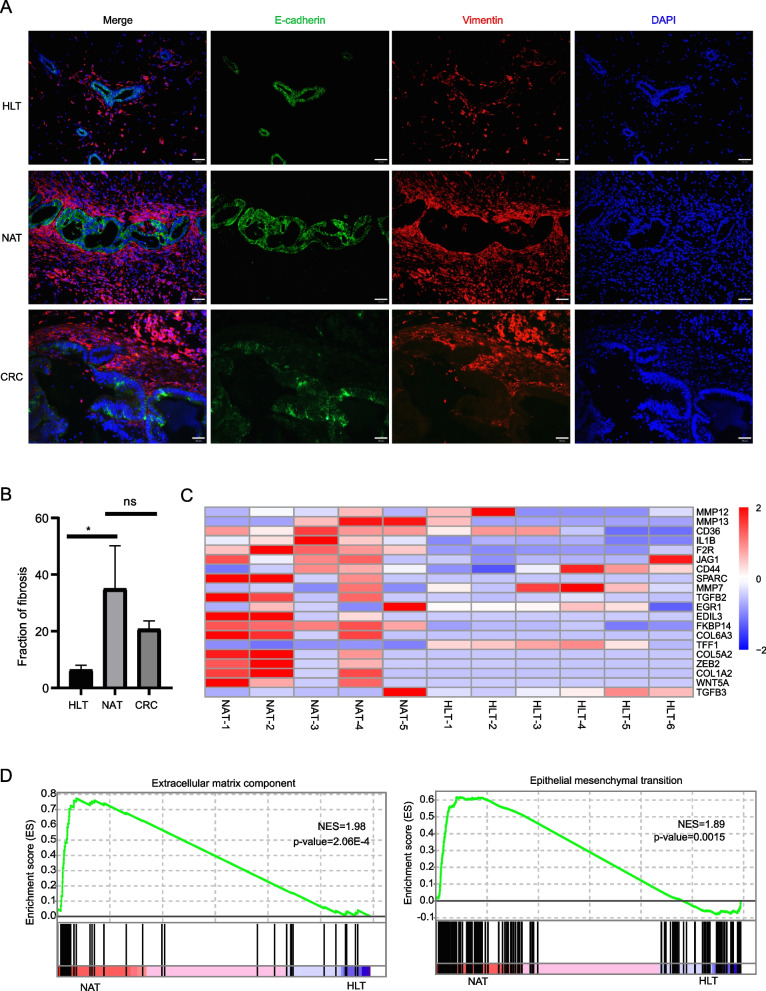
NAT derived clones drive myofibroblast activation. **A** Immunofluorescence micrographs of sections of xenografts formed four weeks after transplantation of clones. Green, E-cadherin; red, Vimentin. Scale bar, 50 μm. **B** Quantification of fibroblast submucosal accumulation in xenografts based on general scale. Data represented as mean ± SD (Student’s t-test). *n* = 3 technical replicates. ∗ *p* < 0.05. **C** Heatmaps of fibrosis related genes among RNA-seq DEGs (FDR < 0.05) of NAT and HLT. **D** Gene set enrichment analysis (GSEA) showing the significantly enriched extracellular matrix component pathway (*p* = 2.06E-4) and EMT pathway (*p* = 0.0015) in NAT-derived clones

Carcinogenesis is typically accompanied by fibrosis, which has been associated with the TGF-β-induced activation of myofibroblasts [28]. We observed that many of the epithelial cysts in the xenografts of clones were surrounded by fibroblast-like cells (Fig. 5A). Vimentin, a key marker of activated myofibroblasts, is expressed by most epithelial cysts in NAT-derived clones, similar to CRC tissue-derived clones, while relatively few of these cysts were present in HLT-derived clones (Fig. 5B; *p* = 0.0311). We also found high expression of fibrosis-related genes in NAT-derived clones compared to HLT-derived clones. Among these fibrosis genes were *TGFB2*, *TGFB3*, *ZEB2* and matrix metallopeptidases (*MMP12*, *MMP13*, *MMP7*; Fig. 5C). In addition, DEGs were primarily enriched in EMT pathways and components of extracellular matrix pathway (Fig. 5D).

### Cocultured HLT-derived clones show high expression of FOSB

**Fig. 6:**
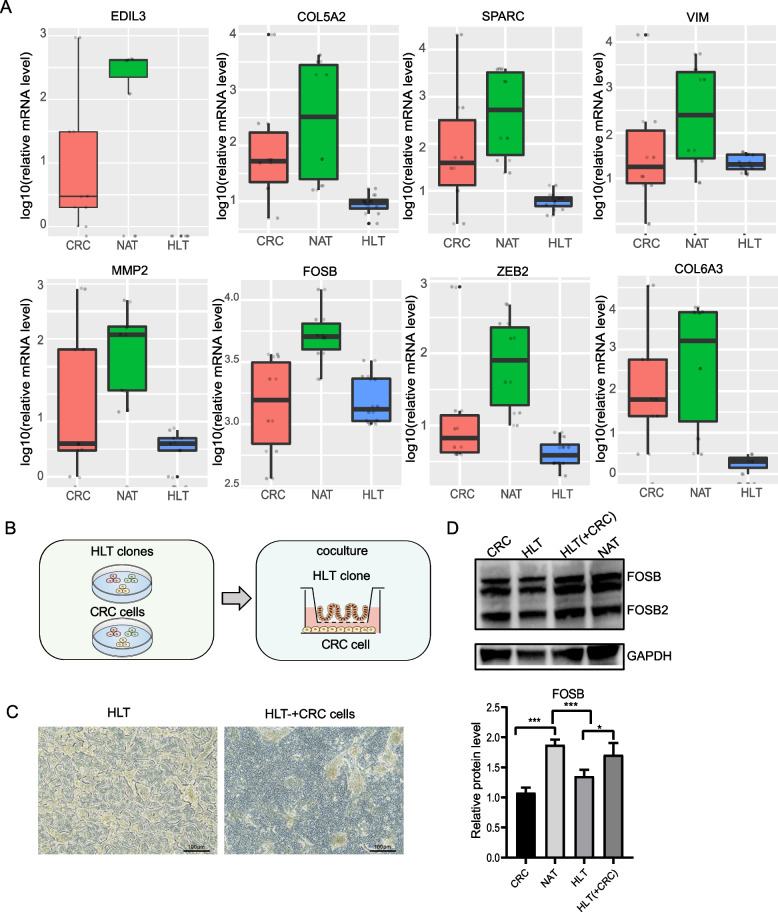
HLT-derived clones cocultured with tumor cells. **A** Genes uniquely expressed in NAT-derived clones compared with HLT and CRC tissues derived clones. **B** Schematic of HLT-derived clones cocultured with tumor cells using the ALI culture system. **C** Representative bright field microscopy images of 3D structures formed by HLT-derived clones when cocultured with tumor cells. Scale bar, 100 μm. **D** Immunoblot analysis showing FOSB expression of HLT-derived clones when cocultured with tumor cells. GAPDH was used as a loading control (left). The band intensity was quantified, data represented the mean ± SD of three independent experiments (right)

In this study, we found a series of genes that were only highly expressed in NAT-derived clones, including *EDIL3*, *COL5A2*, *SPARC*, *VIM*, *MMP2*, *FOSB*, *ZEB2* and *COL6A3* (Fig. 6A). Additionally, we used western blot analysis to confirm these results. Specifically, we found that FOSB was highly expressed in NAT. This gene is rapidly and transiently upregulated in response to external stimuli such as hormones, stress and growth factors [29]. We then explored whether FOSB was upregulated in colonic stem cells derived from HLT that were stimulated by surrounding tumors (Fig. 6B). We plated tumor cells in the upper chamber of transwells and clone cells from HLT in the lower chamber. The factors released between them could pass through the membrane without the cells. The HLT-derived clones formed a highly uniform, 3D serpentine pattern structure that disappeared after coculture with tumor cells (Fig. 6C). For western blot verification, proteins were extracted from the clones after seven days of coculture. The results showed that FOSB was significantly increased in HLT-derived clones after coculture with tumor cells (Fig. 6D).

This finding indicates that colonic stem cells produce stress-associated changes after being stimulated by external tumors, although whether this stimulation will lead to colonic stem cells showing a proinflammatory and profibrotic phenotype requires further research.

## Discussion

The interaction between tumor tissue and the microenvironment exists in the tumor, and the adjacent tissues around the tumor tissue also play a role, such as a direct response to the tumor or an induced response by the tumor [30]. NAT is distinct from both healthy tissue and tumor tissue, which has high TNF-α and TGF-β signaling pathways, as demonstrated by some studies [4]. However, since NAT is made up of a variety of cell types, including immune cells and stromal cells, it is possible that the overall changes in the NAT are the result of changes in these cells. As a result, it is unclear how epithelial cells, including colonic stem cells, are affected. In addition, it is generally accepted that the origin of tumors may be the cancerous of colonic stem cells after accumulating mutations or affected by the microenvironment [11, 31]. Will stem cells derived from NAT also be affected by tumor tissue?

In contrast to colonic stem cells and tumor cells, NAT-derived clones possess a number of distinct, highly expressed genes, as we discovered. Our results revealed NAT-derived clones were changed at the molecular level and were enriched in pathways related to inflammation, including TNF-α signaling pathway, and pathways related to promoting fibrosis, including EMT and TGF-β signaling pathway. Our results also confirmed that NAT-derived clones could recruit inflammatory cells and fibroblasts in mice. The model to study the inflammatory effect of clones by using immunodeficient mice (NSG) here has limitations. First, the restricted host response can be attributed to strain of used in this study, which lacks mature B and T cells, natural killer cells, and shows defects in macrophages and dendritic cells derived from monocytes [27, 32, 33]. We have tested the cell transplantation in immunocompetent mice, but no graft can be formed. It can be carried out in mice with humanized immune system in the future. Second, we only use CD45 to characterize the immune cells recruited in vivo, and the specific type of immune cells recruited need to be further determined. On the other hand, whether this recruitment effect promoted the progression of tumorigenesis or was a protective mechanism is unclear and requires further research.

At the same time, we found that NAT-derived clones have a series of specific and highly expressed genes compared with colonic stem cells and tumor cells. After coculturing colonic stem cells derived from HLT with tumor cells, the expression of FOSB was increased significantly, indicating that this gene may be one of the first genes to respond when surrounding tumor cells release factors [4]. Whether the increase in its expression is related to the inflammatory enrichment and fibrosis pathway in NAT cloning also needs to be further verified.

We hypothesized that tumor cells in patients could impact the surrounding tissue with normal morphological structure. Cytokines and other signaling proteins secreted by tumor cells could cause molecular changes in stem cells in NAT, resulting in the retention of certain memories that can promote inflammation and fibrosis. After removing the influence of tumor tissue, stem cells in NAT maintain their ability to recruit inflammatory cells and fibroblasts. Future clinical treatments must take this into account because it may be one of the factors that leads to cancer recurrence following surgical resection. Destroying this complex molecular network of intercellular communication and signal transduction may be the treatment target of CRC patients.

## Conclusion

We provide new insight into the molecular and functional characteristics of stem cells from NAT in the tumor microenvironment. Our results demonstrate that stem cells could be stimulated by adjacent tumor tissue to undergo changes at the molecular level and produce fibrosis or an inflammatory response. These results suggest that we need to consider the changes in stem cells in NAT in cancer treatment and monitor them in advance.

## Supplementary Information

**Additional file 1.**

**Additional file 2.** 

**Additional file 3: Figure S1.** NAT-derived AQclones can recruit inflammatory cells in vivoAQ.

**Additional file 4.** 

## Data Availability

The sequence data generated in this study has been deposited to the National Genomics Data Center (China National Center for Bioinformation) with accession numbers PRJCA006448.

## Abbreviations

NAT: Normal tissue adjacent to tumor
CRC: Colorectal cancer
HLT: Healthy tissue
ALI: Air–liquid interface
EMT: Epithelial mesenchymal transition
TNF-α: Tumor necrosis factor-α
DEGs: Differentially expressed genes
SCM: Stem cell medium
WES: Whole-exome sequencing
Tic syndrome: Tics and Tourette syndrome
