# Supplementary material for: Colonic stem cells from normal tissues adjacent to tumor drive inflammation and fibrosis in colorectal cancer
Source: Cell Commun Signal. 2023 Aug 1;21:186. doi: 10.1186/s12964-023-01140-1 (PMC10391886; doi:10.1186/s12964-023-01140-1)
Supplement: Supplementary file 4 — Additional file 3: Figure S1. NAT-derived AQclones can recruit inflammatory cells in vivoAQ. [file 12964_2023_1140_MOESM3_ESM.docx]

Figure S1


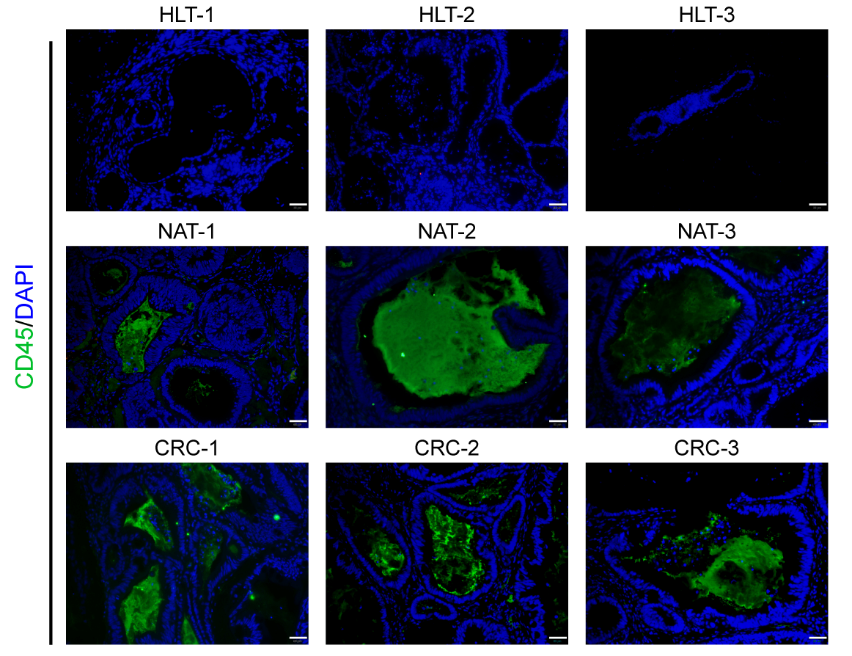


**Figure S1.** **NAT-derived clones can recruit inflammatory cells in vivo.**

Immunofluorescence micrographs of xenografts formed four weeks after transplantation of clones showing the expression of CD45. Scale bar, 50 μm.
