# Supplementary figures and images for: Colonic stem cells from normal tissues adjacent to tumor drive inflammation and fibrosis in colorectal cancer
Source: Cell Commun Signal. 2023 Aug 1;21:186. doi: 10.1186/s12964-023-01140-1 (PMC10391886; doi:10.1186/s12964-023-01140-1)

FOSB

CRC HLT HLT(+CRC) NAT


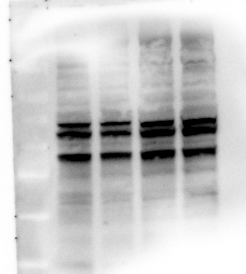


GAPDH

CRC HLT HLT(+CRC) NAT


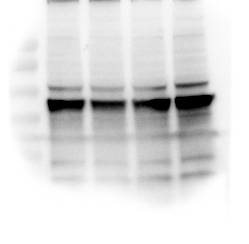

Supplement: Supplementary file 5 — Additional file 4. [file 12964_2023_1140_MOESM4_ESM.docx]
